# Green synthesis and characterization of silver nanoparticles using *Eugenia roxburghii* DC. extract and activity against biofilm-producing bacteria

**DOI:** 10.1038/s41598-022-12484-y

**Published:** 2022-05-19

**Authors:** Alok Kumar Giri, Biswajit Jena, Bhagyashree Biswal, Arun Kumar Pradhan, Manoranjan Arakha, Saumyaprava Acharya, Laxmikanta Acharya

**Affiliations:** 1 Molecular Biology and Genetic Engineering Lab, Centre for Biotechnology, School of Pharmaceutical Sciences, Siksha ‘O’ Anusandhan (Deemed to Be University), Kalinga Nagar, Bhubaneswar, Odisha 751003 India; 2 Department of Nanotechnology, ITER, Siksha ‘O’ Anusandhan (Deemed to Be University) Jagmohan Nagar, Bhubaneswar, Odisha 751030 India

**Keywords:** Biotechnology, Nanoscience and technology

## Abstract

The green synthesis of silver nanoparticles (AgNPs) and their applications have attracted many researchers as the AgNPs are used effectively in targeting specific tissues and pathogenic microorganisms. The purpose of this study is to synthesize and characterize silver nanoparticles from fully expanded leaves of *Eugenia roxburghii* DC., as well as to test their effectiveness in inhibiting biofilm production. In this study, at 0.1 mM concentration of silver nitrate (AgNO3), stable AgNPs were synthesized and authenticated by monitoring the color change of the solution from yellow to brown, which was confirmed with spectrophotometric detection of optical density. The crystalline nature of these AgNPs was detected through an X-Ray Diffraction (XRD) pattern. AgNPs were characterized through a high-resolution transmission electron microscope (HR-TEM) to study the morphology and size of the nanoparticles (NPs). A new biological approach was undertaken through the Congo Red Agar (CRA) plate assay by using the synthesized AgNPs against biofilm production. The AgNPs effectively inhibit biofilm formation and the biofilm-producing bacterial colonies. This could be a significant achievement in contending with many dynamic pathogens.

## Introduction

Most of the plants found in the family Myrtaceae are medicinally important. The secondary metabolites found in these plants can be utilized to cure different diseases. Among the different genera, *Eugenia* is an important taxon in the family having active principles which have pharmaceutical importance. *Eugenia* species produce delicious edible fruits with high vitamin and mineral content. *Eugenia roxburghii* DC. is one such wild edible fruit-producing plant under the family Myrtaceae. It is also known as Roxburgh’s Cherry due to the deliciousness of its fruits. This plant species is mostly found in the coastal and tropical areas of India and Sri Lanka. This species, containing the various secondary metabolites, has anticancer and antibacterial activity^1–7^. Though there is no such systematic study on the medicinal utilization of the species, the plant is used to treat diseases associated with diabetes, arthritis, hypertension, etc., as revealed by the local people^8,9^.

The bioavailability of the active principle is drastically reduced when it is supplied in the form of crude extract, but this can be enhanced when the crude extract is supplied in a modified form like nanomaterial^10^. A nanoparticle (NP) is a microscopic particle having a high surface area. Synthesis of NPs has picked up most attention in recent years due to their vast application in areas like catalysis, optics, electronics^11–13^, antibacterial, and antimicrobial activity^14–16^. The physical and chemical properties of metallic nanoparticles are remarkably different from their corresponding bulk form and can be used as an anti-microbial agent^17^. Plant and plant parts can be used for the reduction of metal to prepare respective metal nanoparticles^18,19^. Among the different metallic NPs, silver nanoparticles (AgNPs) have enormous applications in the medical and biotechnological fields^20^. The synthesis of AgNPs can be achieved both chemically and physically. Physicochemical approaches, on the other hand, include drawbacks such as high running costs, the use of toxic chemicals, and increased energy limits. Physical operations are complex procedures that fail to regulate particle sizes in the nanoscale range. The biggest drawbacks are that they create irregularly sized particles and have a high manufacturing cost^21^. Chemically synthesized NPs are not cost-effective and harm the environment with high energy requirements^22^. This is when biological approaches employing less expensive sources are exploited as AgNPs precursors. The green synthesis of nanoparticles has gained a lot of attraction since it uses non-toxic phytochemicals and avoids the dangerous ingredients that would otherwise be used in chemical synthesis^23^. Green synthesis methods use extracts from diverse plant parts, microbial cells, and biopolymers, and are so classified as such. The nanoparticles created are biocompatible and have the correct level of efficacy for the purpose for which they were created^24^. Metallic NPs can be synthesized biologically using various plants and their extracts which are easily available in huge quantities. The plants and their extracts are safe to handle, less toxic and eco-friendly.

From the leaf extract of *Eugenia jambolana,* silver nanoparticle synthesis was carried out and their phytochemical screening was evaluated^25^. Earlier, reports are available regarding the formation of AgNPs and their biological applications from *Syzygium cumini* ^26^, *Eugenia caryophyllata*^27^. From the leaf extract of *Eugenia uniflora,* silver nanoparticle formation was carried out and their antibacterial and antidiabetic potential were evaluated^28^.

Biofilm is a very fine extracellular polymer fibril that helps the bacteria adhere to the surface^29^. The bacterial community secrets an extracellular polymeric substance after adherence to a matrix or substratum which results in an alternation of phenotype and genetic change with the growth rate^30^. Bacteria forming biofilms possess great resistance to numerous stress conditions including some antibiotics, high salt concentration, acidic conditions, and many oxidizing agents, which results in increasing their pathogenicity^31^. Biofilm formation is seen in most medical devices, catheters, and other implants^32^.

Silver nanoparticle formation has already been reported from different plant extracts such as *Azadirachta indica* (Neem), *Aloe vera*, *Emblica Officinalis* (Amla), *Cinnamomum camphora*^19,33–36^. However, there is no such information about the synthesis of silver nanoparticles and any of their biological applications from the plant *Eugenia roxburghii*. Hence, in this study, an attempt was made to synthesize silver nanoparticles from the leaf extract and their activity against microbes. In our previous study, we found that leaf extract is highly effective in inhibiting the growth of microbes^37^. To enhance the antimicrobial activity of the leaf extract we have tried to prepare AgNPs from the extract to access the effect of the nanoparticles, we have used it to inhibit the growth of biofilms by *S. aureus*. As it has been seen that nanomaterial is better at combating microbes than normal crude extracts, our present investigation will help evaluate the antimicrobial effect of *Eugenia* AgNPs.

## Results

### Characterization by UV–Vis spectrophotometer

**Figure 1:**
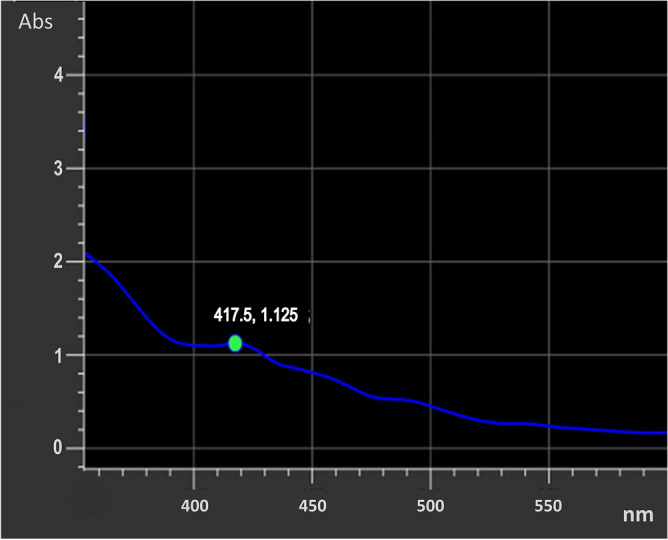
UV–Vis absorption spectra of synthesized AgNPs from *E. roxburghii* leaf extract.

UV–Vis spectrophotometric analysis was carried out for the primary investigation of silver nanoparticle synthesis. A color change has been observed in the mixture of plant extract and AgNPs. The color of the mixture gradually changes from green to yellowish-brown confirming the production of *E. roxburghii* AgNPs. The absorbance of the solution was investigated for one week. From the spectral analysis, it is observed that the AgNPs peak was obtained at 417 nm with the highest peak (Fig. 1) and was stable thereafter for a few days as there was no increase in the absorption.

### Characterization by XRD

**Figure 2:**
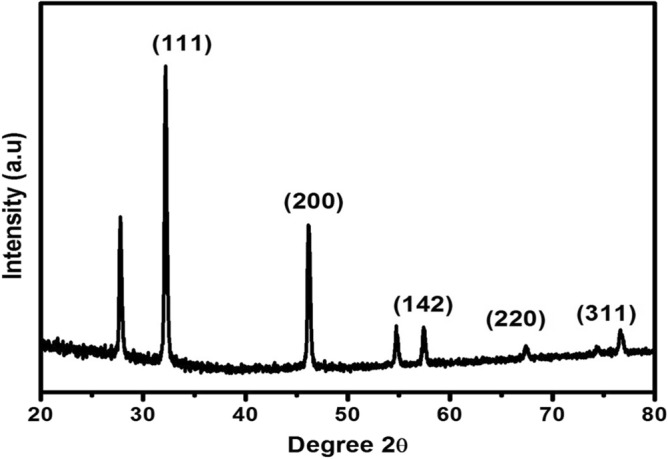
XRD pattern of AgNPs synthesized from *E. roxburghii* leaf extract.

The crystallinity of the synthesized silver nanoparticle using *E. roxburghii* leaf extract was examined through X-ray diffraction (XRD) (Fig. 2). The size of the nanoparticles was calculated based on the Debye–Scherrer equation: (D = kλ/βcosθ).

In the above equation: D represents particle diameter size, K: a constant with a value of 0.9, λ: X-ray source wavelength (0.1541 nm), β and θ represent the FWHM (full width at half maximum), and diffraction angle concerning the (111) lattice planes respectively. The average crystalline size was found to be approximately 35 nm. The lattice parameters for the synthesized AgNPs were determined to be a = 0.4086 nm, b = 0.4086 nm, c = 0.4086 nm respectively. The calculated lattice value was 0.4086 nm, which was nearly identical to the normal lattice parameter of 0.4073 nm for silver^38^.

### Characterization by HR-TEM

**Figure 3:**
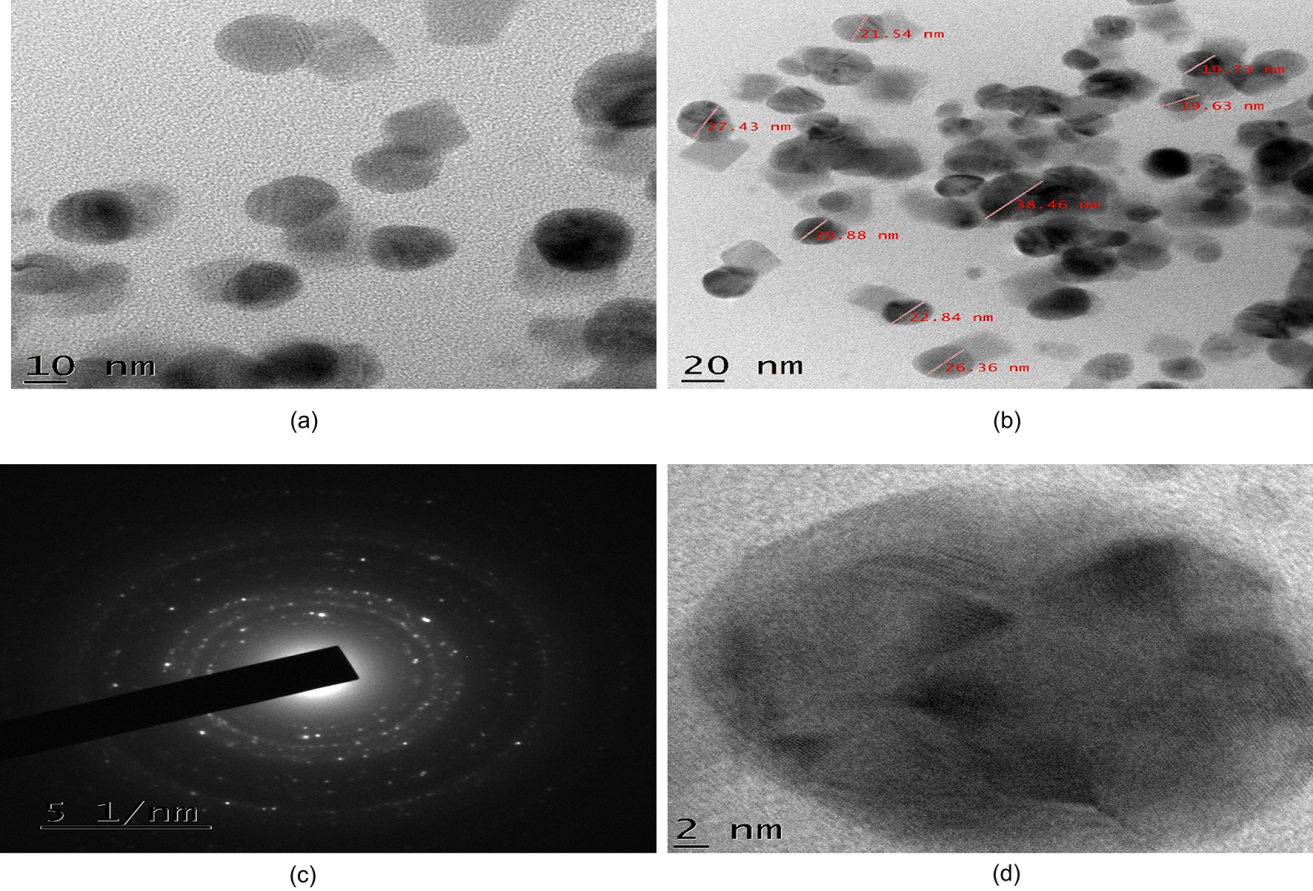
(**a**) TEM micrographs image of the synthesized AgNPs, (**b**) TEM image of different sized AgNPs, (**c**) SAED image of AgNPs, (**d**) HR-TEM image of AgNPs.

The resulted colloidal particles were characterized to determine their shape and size by high-resolution transmission electron microscopy (TEM). For the preparation of the TEM grid, a carbon-coated copper grid was used. A drop of the particle solution was placed over the grid and dried at room temperature. Different TEM micrograph images including SAED pattern and HR-TEM images of the synthesized AgNPs were obtained which are displayed in Fig. 3a–d. The estimated average particle size was approximately 24 nm whereas particle sizes ranged from approximately 19–39 nm.

### Characterization by Zeta sizer

**Figure 4:**
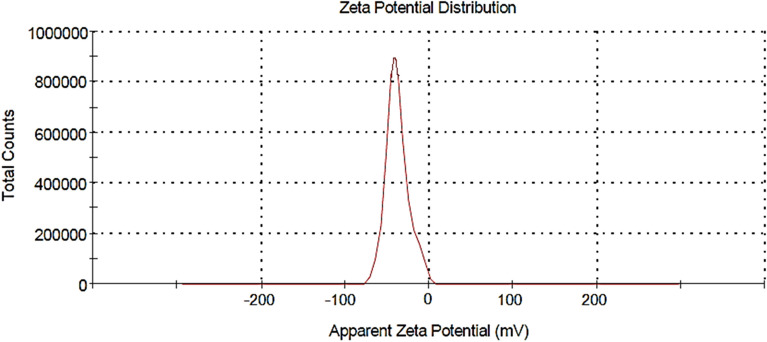
Zeta potential of synthesized AgNPs.

The surface potential of nanoparticles is the potential difference between the medium where nanoparticles are dispersed and the accessible surface of dispersed nanoparticles, which can be analyzed using a zeta sizer. Figure 4 demonstrates the zeta potential of the biosynthesized AgNPs which was found to be  − 37.8 mV. This shows that the AgNPs synthesized from the leaf extract of *E. roxburghii* are highly stable.

### Analysis of antimicrobial activity

**Figure 5:**
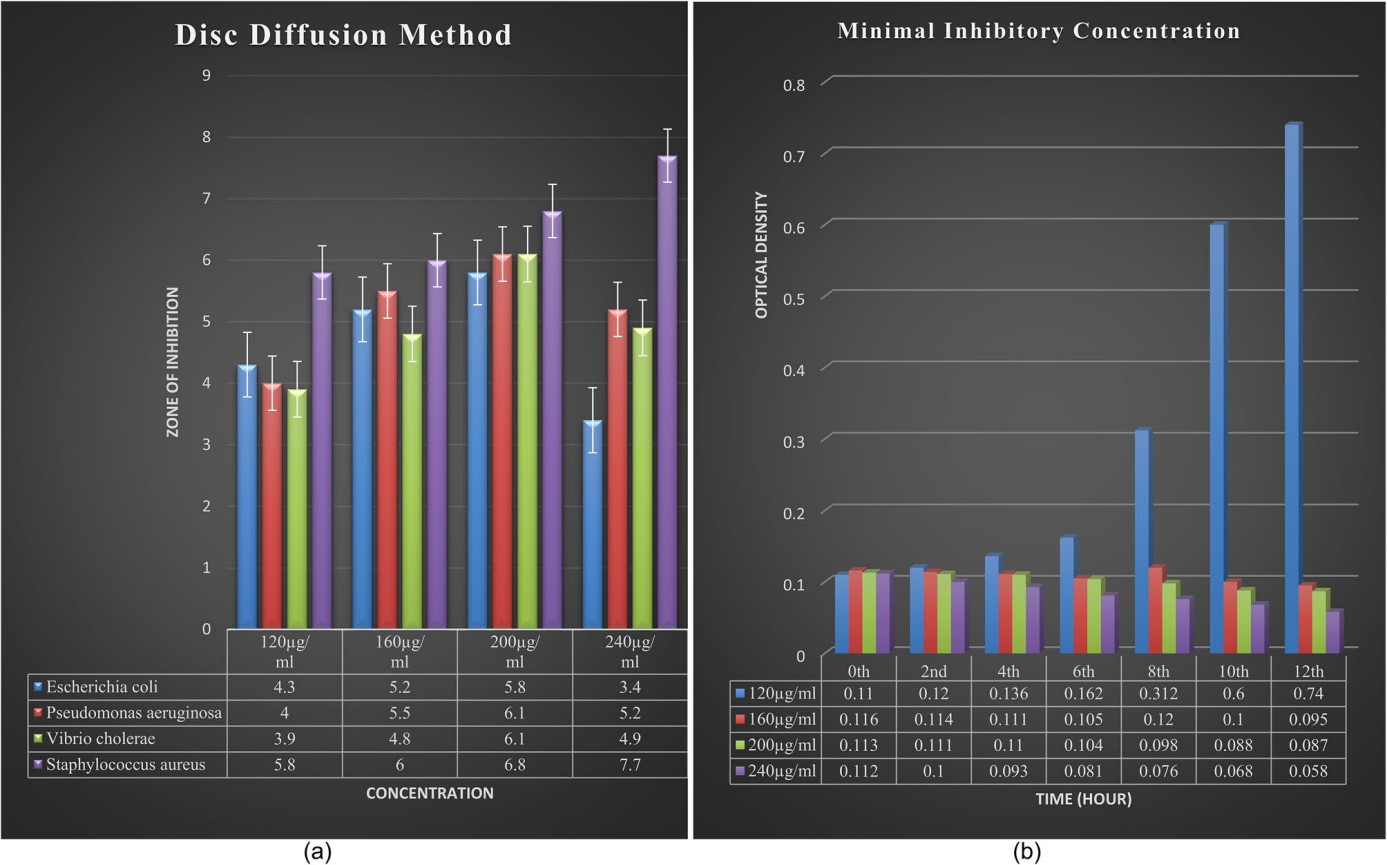
(**a**) Antimicrobial activity test by Disc Diffusion Method against different bacterial strains, (**b**) Minimal Inhibitory Concentration test on *S. aureus.*

An antibacterial activity assay was carried out by using disc diffusion and the MIC method. It was observed that among all the bacteria taken, the AgNPs extract of *E. roxburghii* showed maximum effectiveness towards *S. aureus* (Fig. 5a). So, the MIC experiment was continued with the selection of *S. aureus* bacterium and the MIC test revealed that there was a continuous increase in absorbance at 120 µg/ml concentration whereas, at 240 µg/ml concentration of extract, there was a continuous decrease in absorbance. However, there was no such change in absorbance observed in other concentrations of the extract (Fig. 5b).

### Examine the effect on biofilm

**Figure 6:**
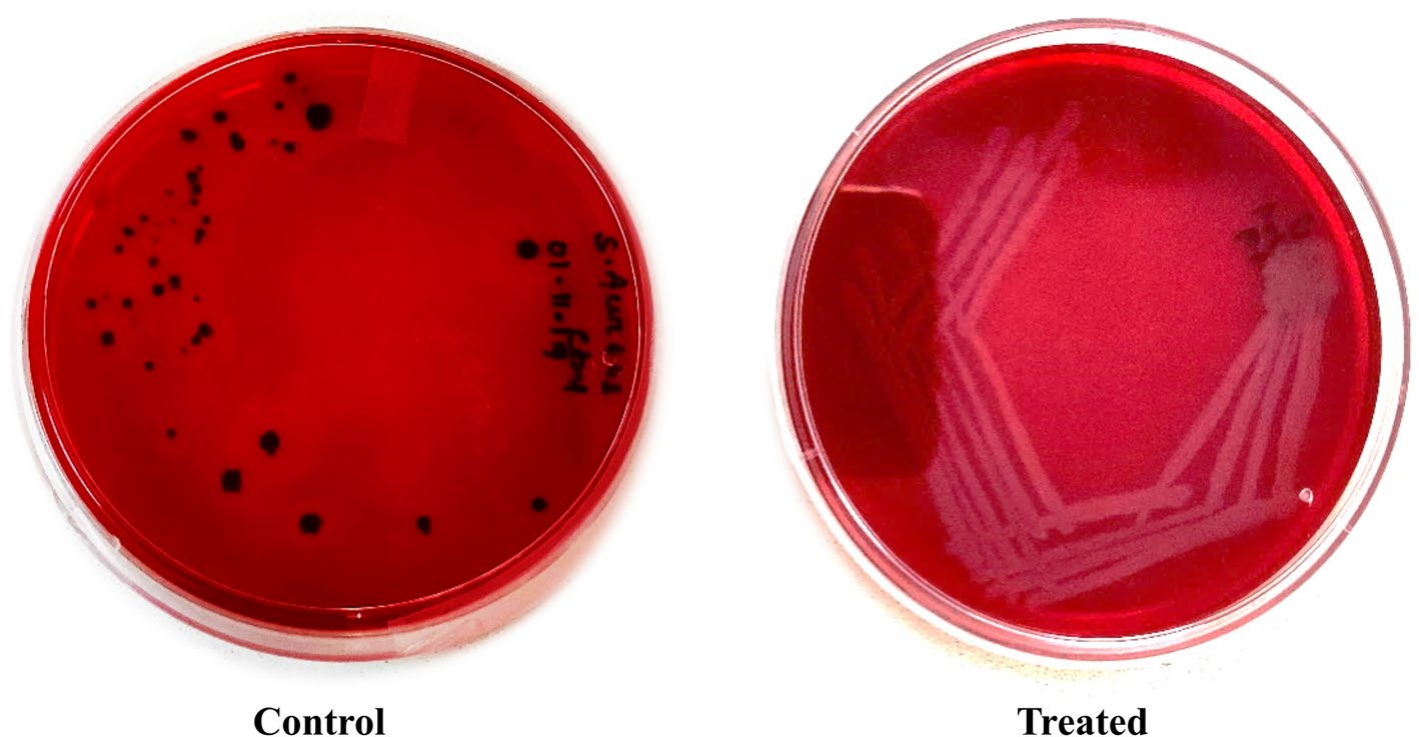
Effect of AgNPs on biofilm production by *S. aureus* on Congo Red Agar plates. *Control = without AgNPs, Treated = With AgNPs.

In this study, it was observed that bacteria changed their color in the control plate (CRA plate without AgNPs) whereas there was no change in the color of bacteria in AgNPs treated CRA plate (Fig. 6). This confirmed the direct inhibition of the biofilm production of bacteria by AgNPs.

## Discussion

The essential enzyme for nitrogen assimilation in a variety of species is nitrate reductase (NR)^39^, which catalyses the conversion of nitrate to nitrite in the cytoplasm of plant cells^40^. An enzymatic pathway involving NADPH-dependent reductase was shown to be responsible for the bioreduction of silver ions. Silver ions exposed to nitrate reductase resulted in the formation of very stable silver NPs and NADPH was found to be the cofactor of the nitrate reductase enzyme^41^. From a previous study, the absorption spectra of synthesized AgNPs from *Syzygium Jambola* was found to be 460 nm and its particle size from TEM analysis was found to be ranging from 6 to 23 nm^42^, similarly in *Syzygium cumini* the UV spectra of synthesized nanoparticle was observed at ~ 450 nm with particle size 3.5 nm from the XRD analysis^43^ and also in *Eugenia uniflora* UV spectra of synthesized nanoparticle was observed at 440 nm having its particle size was ranging from 25 to 50 nm^44^.

In this study, after mixing of extract and silver nitrate solutions a color change of extract was observed over the progression of time which may be due to the reduction of the silver ions leading to the excitation of Surface Plasmon Resonance (SPR) of the AgNPs^45^. To confirm this, UV spectra analysis was carried out and a peak was observed at 417 nm which showed a stable range for nanoparticle formation.

From the XRD pattern of the silver nanoparticle, the structure obtained to be a face-centered cubic one^46^. Four Bragg’s reflections conforming to (111), (200), (220), and (311) planes of metallic silver with FCC crystal structures are understood clearly from the XRD plot (JCPDS No. 89-3722)^47^. So, in the present study, the average crystalline nanoparticle size was measured to be approximately 35 nm. The extra peak obtained at 2θ nearly equal to 28 may be due to the bio-organic phase crystallization over silver nanoparticles surface^48^.

From the resulting images of HR-TEM analysis of synthesized AgNPs, it was observed that there was a presence of few agglomerated AgNPs in some places (Fig. 3a) which may be an indication of further sedimentation. Mostly spherical-shaped particles were observed with variations in their size (Fig. 3b). The average particle size was measured to be approximately 24 nm and the overall particle size ranged between 19 and 39 nm. The electron beam was directed perpendicular to one of the spheres to obtain the SAED (selected area electron diffraction) pattern and the crystallinity of the synthesized AgNO_3_ was confirmed through this pattern (Fig. 3c) which was recorded from one of the nanoparticles. The morphology of a single AgNO_3_ was obtained from the HR-TEM (high-resolution transmission electron microscopy) image and found to be spherical (Fig. 3d).

The antimicrobial activity of silver nanoparticle extract of *E. roxburghii* was tested against four different types of bacteria viz*. E. coli, P. aeruginosa, V. cholera* and *S. aureus.* In the disc diffusion method, the nanoparticle extract showed a significant effect towards *S. aureus* among the above four bacteria for that reason MIC experiment was conducted by taking *S. aureus* bacteria against which different concentrations (120 µg/ml, 160 µg/ml, 200 µg/ml and 240 µg/ml) of nanoparticle extract were treated. In this experiment, while measuring OD, a continuous increase in absorbance at 120 µg/ml concentration of extract may suggest that at a low concentration the bacteria get dominant over the activity of the extract while a continued decrease in absorbance at 240 µg/ml concentration of extract may suggest that at this concentration the extract is efficient enough to remove the bacterial colony.

As the bacteria, *S. aureus* itself is a biofilm-producing bacterium, confirmed biofilm production was observed in the control plate as the plate contains Congo Red media turns into a back color. It was reported that the biofilm-producing capacity of pathogenic bacteria was due to the secretion of exopolysaccharides (EPS)^49^. The change of color from red to black in CRA plates is due to EPS secretion by bacteria^50^. Because of the clinical approach, nowadays biofilm production by the microbes and their growth on the surfaces of medicating instruments and disposable products are the major paths through which microbes enter into the body^51,52^. The biofilms are extremely resistant to host defense mechanisms and also to antibiotic treatment. Adhesion or attachment of microorganisms to a substrate is the first step towards colonization and this strategy has been used for microbial biofilm production^53^. In this study, a new approach was undertaken by synthesizing nanoparticles from biomaterial and using them against biofilm-producing microorganisms to test their effects on them.

## Material and methods

### Preparation of plant extract

Fresh (disease-free) and fully expanded leaves of *Eugenia roxburghii* were collected with the permission of local authorities from the coastal area of Konark, Odisha (latitude 19.878 and longitude 86.101). The plant was taxonomically identified and authenticated by Dr. Laxmikanta Acharya (Associate professor, Centre for Biotechnology, Siksha ‘O’ Anusandhan University, Odisha, India) and a voucher specimen (SOAU/CBT/2020/ER/01) was retained in the department for future reference and the plant has been maintained in an environmentally controlled greenhouse. Experimental research on the plant used for the study complies with relevant institutional, national, and international guidelines and legislation. For the experiment, fresh and healthy leaves were taken and washed three times with distilled water. After washing, the methanolic extract was prepared by finely grinding 25 g of leaves with liquid nitrogen in a mortar and pestle followed by the addition of 250 ml of methanol. The debris from the leaf extract was separated with filter paper (Whatman No 1). The filtrate was collected and preserved at − 20 °C.

### Preparation of AgNPs with Eugenia roxburghii leaf extract

**Figure 7:**
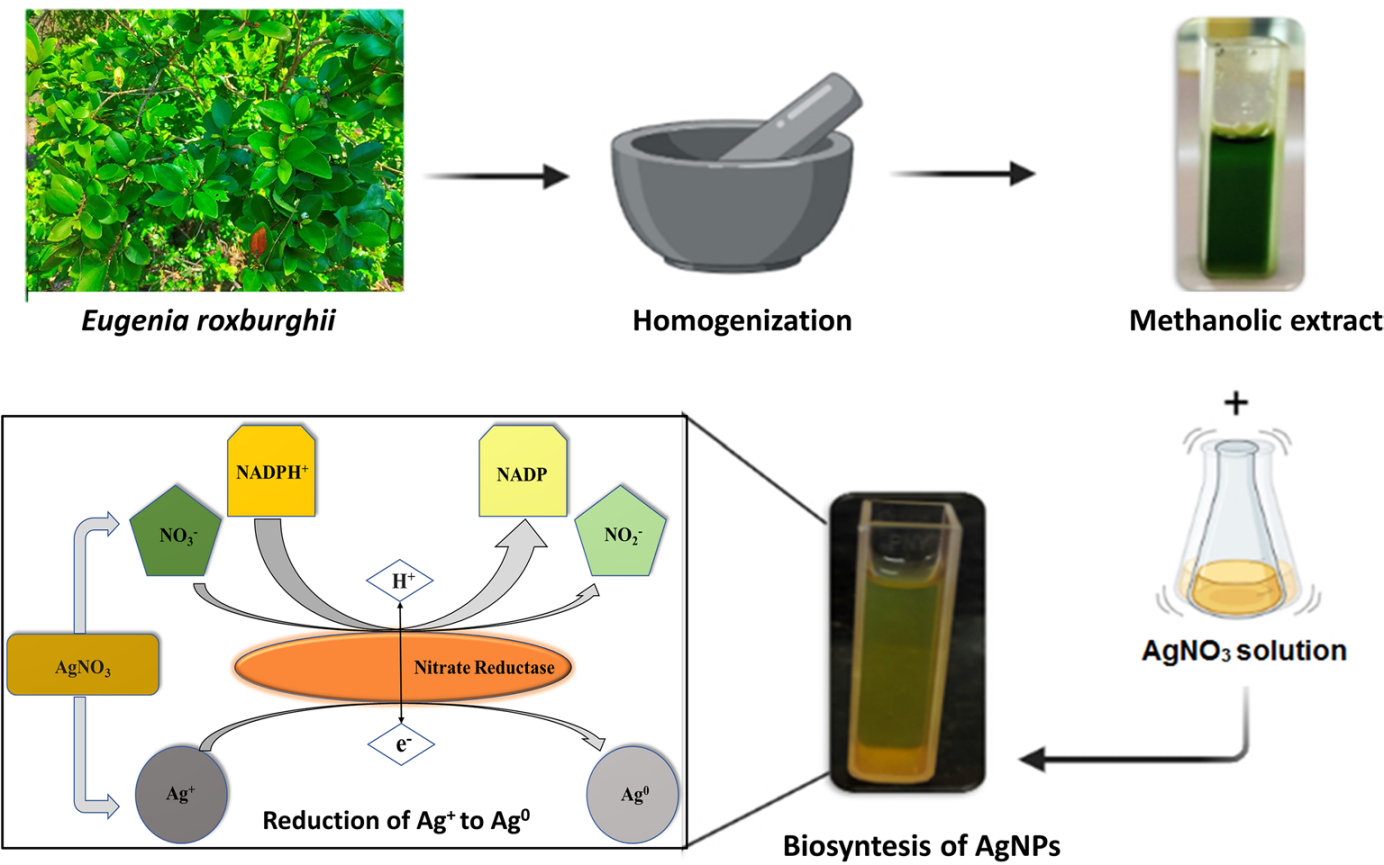
Synthesis of AgNPs from *E. roxburghii* leaf extract.

One molar silver nitrate (AgNO_3_) stock solution was prepared. From that stock solution, 0.1 mM AgNO3 solution was taken along with the leaf extract in a 5:1 proportion for the preparation of AgNPs. 20 ml of leaf extract was mixed with 100 ml of 0.1 mM AgNO_3_ solution and incubated in a shaker incubator at 300 rpm at 37 °C for 48 h. Gradually the deep green color solution changed to yellowish-brown color which indicated the conversion of Ag^+^ to Ag^0^ (Fig. 7). The effect of this synthesis of silver nanoparticles was monitored in UV–VIS spectrophotometer. The spectrophotometric reading was taken at different time intervals.

### Characterization of AgNPs

Various analytical techniques were used for the characterization of green synthesized silver nanoparticles from *E. roxburghii* leaf extract. Constant monitoring of the reaction for the reduction of Ag^+^ ion by taking the OD (optical density) from 200–700 nm in a double beam UV–Vis spectrophotometer (Hitachi, UH5300). Further characterization of AgNPs was carried out through XRD (Rigaku, Ultima IV, Japan) equipped with Cu-Kα radiation; a crystal monochromator employing wavelengths of 0.1541 nm in a 2θ range from 20° to 80°. HR-TEM analysis of derived nanoparticles was carried out on a JEM 2100 (Jeol), operated at 200 kV.

### Antimicrobial activity test

#### By disc diffusion method

To check the antimicrobial activity of *E. roxburghii* AgNPs extract, a disc diffusion method was carried out. For this test, different strains of bacteria such as *E. coli* (ATCC-443)*, P. aeruginosa* (Clinically isolated from SCB medical college, Microbiology department, Cuttack, Odisha, India)*, V. cholera* (ATCC-3906), and *S. aureus* (ATCC-96) were used which were identified and confirmed at the Centre for Biotechnology, Siksha O’ Anusandhan (Deemed to be University), Odisha, India. Active bacterial cultures were revived by inoculating a loop full of bacterial culture in nutrient broth from the stock maintained at 4 °C and incubated overnight at 37 °C in a shaker incubator at 800 rpm. Nutrient agar plates were prepared and spreading of 60 µl of each bacterial culture was carried out. Different concentrations such as 120 µg/ml, 160 µg/ml, 200 µg/ml, and 240 µg/ml of AgNPs extract infused discs were prepared and placed over the bacterial spread plates followed by incubation overnight at 37 °C. the observed zone of inhibition was measured in mm against the commercially available antibiotic ciprofloxacin.

#### By minimal inhibitory concentration (MIC)

To evaluate the minimal inhibitory concentration, different concentrations (120 µg/ml, 160 µg/ml, 200 µg/ml and 240 µg/ml) of AgNPs extract were tested against *S. aureus.* For this experiment, 25 ml of nutrient broth was added to four different conical flasks containing different concentrations of the AgNPs extract mentioned above followed by the addition of 100 µl of bacterial culture. After the addition of bacterial culture, OD was measured at 600 nm in every 2 h interval of time from 0 to 12 h followed by incubation at 37 °C at 800 rpm.

#### Effect of AgNPs on Biofilm synthesis

In this study, the main target was to evaluate the effectiveness of AgNPs extract of *E. roxburghii* against biofilm production and this experiment was carried out by selecting the *S. aureus* bacterium. A Congo Red Agar (CRA) plate assay was carried out to investigate the activity of AgNPs on Biofilm production^54^. Two media plates named control and treated were taken in which the control plate was incorporated with Congo Red Dye mixed nutrient agar streaked with *S. aureus* bacteria and the treated plate was incorporated with a mixture of nutrient agar, Congo Red Dye, and AgNPs extract (0.065 g/ml) streaked with *S. aureus*. Then the plates were incubated at 34 °C for 3 days.

## Conclusion

The silver nanoparticle prepared from *E. roxburghii* leaf extract were observed under UV–Vis Spectroscopy monitored at 417 nm and their crystallinity nature was confirmed from their XRD study. AgNPs are found to be very effective against biofilm production by bacteria. However, an experiment must be carried out to find the effect of the NPs on the animal model as well as on human beings for the evaluation of efficacy. Toxicological studies are also required to eradicate any kind of intoxication in a mouse model or human being. Once the NP is found nontoxic or safe in vivo studies, the nanoparticle can be utilized for the treatment of various diseases such as diabetes, arthritis, hypertension, etc. AgNPs play a major role in inhibiting bacterial colonies and biofilm formation. This study springs a new approach for synthesizing nanoparticles from the leaves of *E. roxburghii* which is found out to be inhibiting biofilm production and bacterial colonies can be a significant achievement in contending many dynamic pathogens. Other nanoparticles besides AgNPs can also be prepared from the leaf extract and their medicinal properties can be exploited for the remedy of various diseases. So, the present work can be considered an attempt to exploit the active principle present in the leaf of *E. roxburghii* to cure various ailments.

## Abbreviations

AgNPs: Silver nanoparticles
NPs: Nanoparticles
AgNO_3_: Silver nitrate
XRD: Xray Diffraction
TEM: Transmission Electron Microscope
SAED: Selected Area Electron Diffraction
FWHM: Full Width at Half Maximum
FCC: Face Centered Cubic
CRA: Congo Red Agar
EPS: Exopolysaccharide
Units: G, ml, M
